# Surgical Management of an Advanced Case of Invasive Cervical Root Resorption: A Case Report

**DOI:** 10.7759/cureus.93447

**Published:** 2025-09-28

**Authors:** Rajashree Tamuli, Geeta Asthana, Sadhna Manglani, Ragini Kulkarni, Anooja Mathirat

**Affiliations:** 1 Department of Conservative Dentistry and Endodontics, Government Dental College and Hospital, Ahmedabad, IND

**Keywords:** bioceramic sealer, biodentine, cbct, external cervical root resorption, trichloroacetic acid

## Abstract

External cervical resorption (ECR) is a pathological condition characterized by progressive loss of tooth structure, often remaining asymptomatic until reaching an advanced stage. The condition can have a multifactorial origin, with possible contributing factors such as trauma, orthodontic movement, and microbial activity. Early diagnosis through advanced imaging techniques such as cone-beam computed tomography (CBCT) is crucial for optimal treatment planning and prognosis.

This case report describes the surgical management of a severe ECR lesion affecting a maxillary anterior tooth in a 45-year-old female who presented with pain and localized swelling. Clinical and radiographic evaluation revealed an invasive cervical resorptive lesion with pulpal involvement. Surgical intervention included meticulous removal of resorptive tissue, chemical cauterization using 10% trichloroacetic acid for decontamination, and restoration of the defect with Biodentine (Septodont, St Maur-des-Fossés, France). Following root canal therapy and definitive restoration, the patient was reviewed periodically. At the one-year follow-up, she remained asymptomatic with no clinical or radiographic signs of recurrence. The Biodentine restoration exhibited excellent adaptation, and CBCT confirmed successful treatment with no further resorptive progression.

## Introduction

External cervical resorption (ECR) is an aggressive form of invasive root resorption that originates at the enamel-cementum junction. If left untreated, it can cause extensive destruction of tooth structure, eventually compromising the pulp, periodontal apparatus, and periapical tissues, ultimately resulting in tooth loss [1-4]. Numerous predisposing factors have been proposed, such as traumatic injuries, orthodontic forces, parafunctional activity, occlusal disharmony, intracoronal bleaching, poor oral hygiene, periodontal therapy, developmental or eruption anomalies, and certain viral infections [2,5-7].

Epidemiological studies report the prevalence of ECR between 0.02% and 2.3% [8,9], underscoring its rarity yet significant clinical relevance. In simpler terms, ECR is a condition in which the root surface near the cervical gingiva is gradually resorbed, often silently, until symptoms appear. Such complexity makes early diagnosis critical.

Although the exact etiology and pathogenesis of ECR remain incompletely understood, inflammation is regarded as an essential factor in its initiation [10]. One prevailing hypothesis suggests that the process begins when the protective cementum layer, situated just apical to the epithelial attachment, is absent or compromised [11,12]. This defect may be attributed to a developmental discontinuity at the cemento-enamel junction (CEJ), or to traumatic injury affecting the precementum. Once the root dentin is exposed, it becomes vulnerable to colonization by clastic cells, triggering the onset and progression of resorption [5,13].

ECR is a dynamic and progressive condition in which exposed dentin is gradually infiltrated by clastic cells and replaced with non-infective, hyperplastic invasive fibrovascular tissue [13]. Concurrently, areas of apposition and remodeling of bone-like tissue can occur, giving the process both destructive and reparative characteristics [14]. Resorptive lesions often present with one or multiple portals of entry into the dentin, while the pulp is initially safeguarded by a peri-canalar resorption-resistant sheet (PRRS) - a protective complex of odontoblasts and predentin - that slows the lesion’s progression toward the root canal [14,15]. In advanced stages, however, the resorptive activity breaches the PRRS, leading to pulpal inflammation, pulp necrosis, and, if left untreated, concomitant periodontal breakdown and eventual tooth loss.

The management of ECR is widely recognized to rely on addressing the patient’s primary symptoms while accurately assessing the size, location, and accessibility of the lesion [6,16]. Treatment strategies are tailored to these factors and can vary considerably. According to the European Society of Endodontology Position Statement [7], available options include external repair, internal repair, intentional replantation, periodic monitoring, and extraction. More recent literature has similarly outlined a spectrum of approaches, ranging from conservative repair methods to palliative care and tooth removal [17].

This case report describes an uncommon clinical presentation of an advanced case of external invasive cervical root resorption, successfully managed using a combined surgical and restorative approach.

## Case presentation

A 45-year-old female patient presented to the Department of Conservative Dentistry and Endodontics with a chief complaint of pain and swelling in the upper front teeth region. The patient reported experiencing intermittent discomfort and noticeable swelling in the gum around the upper right central incisor for the past two months. The swelling had gradually increased in size over the past two days, accompanied by occasional bleeding upon brushing and discomfort when chewing. The patient’s medical history was non-contributory, with no known systemic conditions or allergies. However, she reported no previous trauma or history of orthodontic treatment.

**Figure 1:**
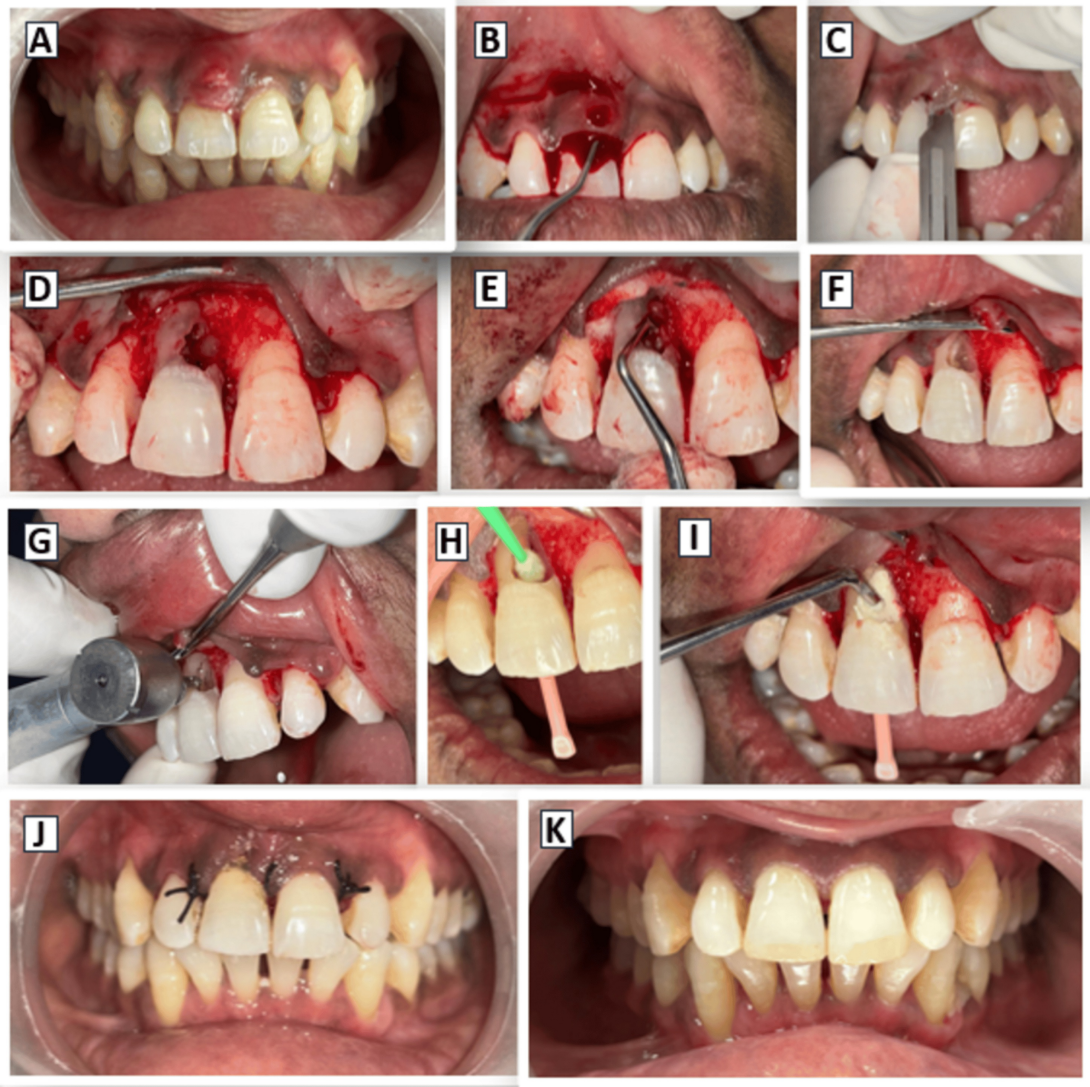
(A) Pre-operative photograph, (B) Curettage, (C) Placement of incision, (D) Flap reflection, (E-G) Removal of granulation tissue, (H) Application of TCA, (I) Condensation of Biodentine, (J) Immediate post-operative photograph with suture placement, (K) One-year follow-up photograph. TCA, Trichloroacetic Acid

**Figure 2:**
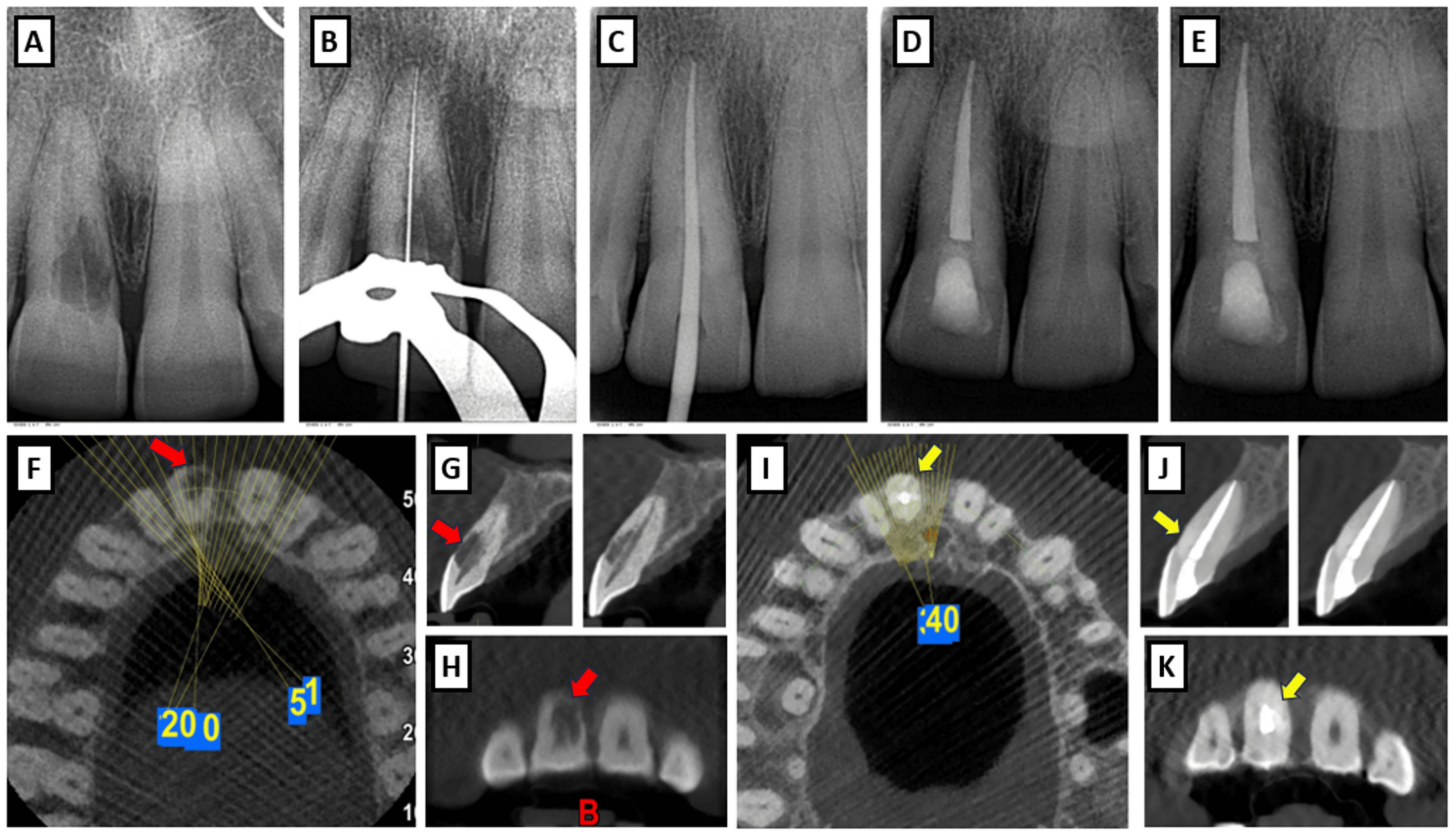
(A) Pre-operative radiograph, (B) Working length radiograph, (C) Master cone radiograph, (D) Immediate post-operative radiograph, (E) One-year follow-up radiograph, (F-H) Pre-operative CBCT images - axial, sagittal, and coronal sections with a slice thickness of 0.5 mm (red arrow denotes the resorption areas), (I-K) Post-operative CBCT images - axial, sagittal, and coronal sections (yellow arrow denotes the intact Biodentine restoration with no further progression of resorption). CBCT, Cone-Beam Computed Tomography

Upon clinical examination, tooth #11 exhibited localized gingival swelling at the cervical third, along with the presence of a deep periodontal pocket (6 mm in depth) and bleeding on probing. The tooth had a mild degree of mobility (Grade I), and the surrounding gingiva was erythematous (Figure 1A). No caries or fractures were evident. Percussion and palpation tests revealed mild discomfort, and the tooth was non-vital, as confirmed by thermal and electric pulp testing. There were no signs of systemic involvement or generalized inflammation. Radiographic examination revealed an irregularly bordered, mottled radiolucency involving the cervical region of the root of tooth #11, consistent with ECR (Figure 2A). Unlike the classic “pink spot” discoloration typically seen in ECR, this case presented atypically with gingival swelling and pain, initially mimicking periodontal or periapical pathology. To further confirm the extent of the lesion, cone-beam computed tomography (CBCT) was performed, which revealed that the defect extended to the middle third of the root, with a circumferential spread of 90 degrees and pulpal involvement (Figures 2F-2H). Therefore, the resorptive lesion was classified as a Heithersay’s Class IV ECR defect (an extensive resorptive defect extending beyond the coronal third of the root) and a Patel’s 3Ap ECR defect (where "3" indicates extension into the middle third of the root, "A" indicates circumferential spread of 90 degrees, and "p" indicates pulpal involvement) (Table 1). No periapical pathology was noted.

**Table 1:** Patel’s classification of external cervical resorption. d stands for dentine, and p stands for pulpal involvement.

Height	Circumferential spread	Proximity to the root canal
At the cemento-enamel junction level (supracrestal)	A ≤ 90°	Lesion confined to dentine (d)
Extends into the coronal third of the root (subcrestal)	B > 90° and ≤ 180°	Probable pulpal involvement (p)
Extends into the mid-third of the root	C > 180° and ≤ 270°	-
Extends into the apical-third of the root	D > 270°	-

The lesion was identified as active and progressive, making immediate intervention necessary to halt the resorptive process and preserve the tooth. Given the location and extent of the resorptive lesion, a surgical approach was planned. The patient was informed of the proposed treatment plan and possible outcomes, and written informed consent was obtained.

Clinically, the lesion presented with gingival swelling and signs of active inflammation. In this condition, direct flap reflection posed a risk of flap tearing due to friable tissue, potentially leading to gingival recession and compromised esthetics postoperatively in the anterior region, which could affect the esthetic outcome after healing. To mitigate this risk, an initial phase of periodontal curettage was undertaken to reduce inflammation and ensure safe, controlled flap reflection, without soft tissue damage during the subsequent surgical procedure (Figure 1B).

The patient was recalled after one week for the surgical procedure. Prior to surgery, endodontic therapy was initiated. Under rubber dam isolation, access preparation was performed using an Endo Access Bur, followed by an Endo-Z tungsten carbide bur (Dentsply Maillefer, Ballaigues, Switzerland). Working length was established (Figure 2B), and the canal was prepared using the ProTaper Gold rotary file system to an apical size of F5 (Dentsply Maillefer, Ballaigues, Switzerland). The canal was irrigated with 10 mL of 3% sodium hypochlorite between each instrument, using a 30-gauge open-ended irrigation needle positioned 3 mm short of the working length to minimize the risk of apical extrusion of the irrigant. After confirming the fit of the corresponding gutta-percha cone, it was placed within the canal to maintain patency throughout the surgical procedure (Figure 2C).

The surgical procedure was performed under local anesthesia using 2% lidocaine with 1:80,000 epinephrine. Under magnifying loupes (3×), a full-thickness mucoperiosteal flap was carefully elevated to provide adequate access to the resorptive defect (Figure 1C). On exposure, the lesion was identified at the cervical third of the root, presenting as an irregular defect with evident soft tissue ingrowth consistent with active resorption (Figure 1D). A combination of manual curettes, sharp excavators, and rotary instruments was used to carefully remove the granulation tissue and clean the root surface. The ragged margins of the resorptive cavity were then smoothened using a round carbide bur to create a clean, well-defined outline for restoration (Figures 1E-1G).

After complete debridement of the resorptive tissue, the root surface was disinfected with a 90% aqueous solution of trichloroacetic acid (TCA). A microbrush was dipped into TCA and then gently pressed to remove excess liquid before carefully applying it to the resorptive site. The areas immediately adjacent to the defect were protected with the application of glycerol to prevent inadvertent contact with TCA. It was then allowed to remain in contact with the exposed root surface for three minutes, following Heithersay’s protocol (Figure 1H), and was thoroughly rinsed off with sterile saline to ensure the removal of any remaining resorptive cells and debris, which is essential for preventing reinfection. Finally, the exposed tooth surface was refreshed with a round carbide bur before final restoration, to ensure proper bonding of the restorative material.

For defect restoration, Biodentine (Septodont, St Maur-des-Fossés, France) was chosen. The material was prepared in accordance with the manufacturer’s recommendations, inserted into the cavity, and carefully adapted to reproduce the natural root contour (Figure 1I). After ensuring the material had set, the flap was repositioned and sutured to achieve primary closure.

The canal was again irrigated with 10 mL of 3% sodium hypochlorite, 10 mL of saline, followed by 5 mL of 17% EDTA, and dried with absorbent paper points. A master cone radiograph was taken (Figure 2C), and obturation was completed with a single cone technique using bioceramic sealer, BioActive RCS (SafeEndo, Vadodara, India) (Figure 2D). The access was restored with glass ionomer cement (Type II GC-Gold Label, GC Corporation, Tokyo, Japan) and a final restoration with composite (Ivoclar Vivadent, Schaan, Liechtenstein). Postoperative instructions were given, and the patient was scheduled for follow-up visits at one week, one month, six months, and one year after the surgery.

At the one-year follow-up, the patient remained asymptomatic, and the gingival tissues around tooth #11 were healthy (Figure 1K). Radiographic assessment showed complete resolution of the radiolucent lesion, and the Biodentine filling remained well adapted to the root surface (Figure 2E). On CBCT examination, no further resorptive activity was observed (Figures 2I-2K).

## Discussion

The clinical management of ECR aims to retain the affected teeth in a healthy, functional and esthetic state [10].

In the present case, the clinical presentation was that of a localized gingival swelling, accompanied by pain, initially mimicking a periodontal defect or periapical pathology. This is an unusual finding when compared to most cases reported in the literature, where ECR typically manifests as a pinkish discoloration of the crown due to underlying resorptive changes. A probable etiological factor in this case could be the presence of a developmental gap at the CEJ, which may have exposed the underlying root dentine to clastic cell activity, thereby initiating and propagating the resorption process [1].

The gingival swelling observed in this case can be attributed to the chronic inflammatory response elicited by the resorptive process. As the lesion progressed, bacterial infiltration and breakdown of surrounding periodontal tissues may have likely triggered localized inflammation, resulting in granulation tissue formation and consequent swelling. This inflammatory enlargement masked the underlying resorptive defect, further contributing to its atypical clinical presentation.

To facilitate diagnosis, treatment planning, and prognosis assessment, several classification systems for ECR have been described in the literature. Heithersay’s clinical classification (1999) categorizes lesions into four classes based on clinical and radiographic findings: Class 1 - small, well-defined lesion near the cervical area; Class 2 - well-defined lesion close to the coronal pulp with minimal radicular extension; Class 3 - deeper invasion into coronal and radicular dentin up to the coronal third of the root; and Class 4 - large lesion extending beyond the coronal third of the root, often involving multiple areas. The classification was based on conventional periapical radiographs [3]. More recently, both the European Society of Endodontology (ESE) and the joint position statement of the American Association of Endodontists with the American Academy of Oral and Maxillofacial Radiology (AAE/AAOMR) have emphasized that periapical radiographs frequently underestimate the true nature of ECR when compared with CBCT. Using CBCT, information regarding the true ECR size, location, circumferential spread, proximity to the root canal, and accessibility can be gained, which is crucial to determine an appropriate treatment plan. In 2018, Patel et al. created a new three-dimensional classification for ECR based on CBCT that evaluates lesion height (1-4), circumferential spread (A-D), and proximity to the pulp (d/p). This system allows more precise assessment of lesion boundaries, extent, and pulp involvement, thereby aiding in the formulation of minimally invasive and targeted treatment strategies [6,7].

The management and prognosis of ECR depend on the extent of the lesion, the vitality of the tooth, and its potential for restoration [17]. In the present case, the resorptive defect was extensive and became clearly visible after flap elevation. Given the significant involvement of the labial tooth structure, thorough debridement of the resorptive tissue was crucial to ensure a successful outcome. To facilitate this, surgical intervention was undertaken in conjunction with debridement using a 90% aqueous solution of TCA, to ensure thorough disinfection prior to restoration. TCA, in this concentration, was considered the preferred choice due to its ability to promote coagulation necrosis of hyperplastic invasive tissue and penetrate even the most inaccessible resorptive channels communicating with the periodontal ligament. This deep penetration helps to cauterize residual tissue and reduce recurrence risk. However, a potential limitation is the risk of accidental damage to adjacent soft tissues, which was mitigated in this case by the application of glycerol as a protective barrier. In addition, dentine conditioned with TCA undergoes deep demineralization, which may negatively affect adhesion; hence, the conditioned root surface was refreshed with a round carbide bur before final restoration [1].

The duration of TCA application also plays a decisive role in its clinical efficacy. Literature suggests that an application period of approximately three to four minutes is optimal, as this allows sufficient time for TCA to induce complete coagulation necrosis of resorptive tissue, while avoiding excessive penetration that could compromise adjacent healthy periodontal ligament or pulpal tissues [18]. This controlled timing, combined with careful isolation, ensures a predictable and safe outcome.

In the present case, Biodentine (Septodont, Lancaster, PA, USA) was utilized to repair the resorptive defect. Biodentine, composed of tricalcium silicate, calcium carbonate, and calcium chloride, is a bioactive material with superior sealing properties, biocompatibility, and favorable handling characteristics. Its capacity to promote periodontal ligament reattachment, stimulate dentin-like tissue formation, and provide an effective seal for resorptive defects renders it a highly suitable material for the management of ECR [19]. Biodentine’s compressive strength, which is comparable to that of natural dentin, along with its rapid setting time, further supports its suitability for use in the present case [20].

To contextualize the present report, similar cases of ECR have been described in the literature, with varying presentations, classifications, and treatment modalities. A comparative overview is presented in Table 2, which shows that, while different biomaterials and protocols have been used, outcomes are generally more favorable when lesions are detected early and treated comprehensively. The present case is distinct in its atypical presentation as a gingival swelling, rather than the more common “pink spot” discoloration, yet it achieved stable clinical and radiographic outcomes with Biodentine at one-year follow-up.

**Table 2:** Comparison table listing available cases of external cervical resorption published in the literature. This table summarizes documented cases of ECR, highlighting variations in etiology, clinical presentation, diagnostic imaging modalities, and treatment approaches, as reported in the literature. CBCT, Cone-Beam Computed Tomography; ECR, External Cervical Resorption; ESE, European Society of Endodontology; GIC, Glass Ionomer Cement; MTA, Mineral Trioxide Aggregate; RMGIC, Resin-Modified Glass Ionomer Cement; TCA, Trichloroacetic Acid

Author/year	Study type	Cases/teeth	Classification used	Lesion extent (typical)	Management approach	Cauterization/Disinfection	Restorative material	Endodontic approach	Follow-up duration	Outcome summary
Heithersay (1999) [2]	Case series	81 teeth (various classes)	Heithersay Class I-IV	Varies by class	Surgical debridement + TCA; restoration	TCA ~90% (≈3-4 min)	GIC/MTA	As indicated (when pulp threatened/involved)	Months-years (varied)	Higher success in Class I-II; poor for Class IV
Heithersay (2004) [3]	Narrative review	-	Heithersay Class I-IV	-	Reiterates surgical debridement + TCA protocol	TCA ~90%	GIC/MTA (contextual)	Case-dependent	-	Protocol rationale and prognosis by class
Schwartz et al. (2010) [4]	Case series (3 practices)	Multiple cases	Not specified	Cervical defects	Surgical repair	Not specified	Resin‑modified GIC	Case-dependent	Varied	Mixed outcomes; some recurrence
Espona et al. (2018) [16]	Case report (anterior zone)	Single case	Heithersay Class III	Coronal + radicular dentin	Flap surgery + debridement	Not specified (standard debridement)	MTA	As indicated	≈2 years	Stable clinical and radiographic results
Patel et al. (2018) (ESE Position) [7]	Position statement	-	CBCT‑based (Patel 3D) recommended	Height, circumferential spread, pulp proximity	External repair, internal repair, replantation, monitoring, extraction	Case-dependent (e.g., TCA)	Case-dependent (MTA/Biodentine/RMGIC)	Guided by pulp proximity	-	CBCT improves planning and prognosis estimation
Chen et al. (2021) [17]	Review	Multiple reports	Heithersay & CBCT-based	Varies	Conservative to surgical; extraction when extensive	Often TCA or meticulous debridement	MTA/Biodentine/RMGIC	As indicated by pulp status	Varies	Prognosis depends on size, access, and pulpal proximity

## Conclusions

The present case highlights the importance of thorough clinical and radiographic evaluation in diagnosing atypical presentations of ECR. While most cases in the literature present with the characteristic “pink spot” discoloration, this case was distinguished by its unusual manifestation as a localized gingival swelling with pain, initially mimicking periodontal or periapical pathology. Such an atypical profile poses a diagnostic challenge and reinforces the need for heightened clinical vigilance. The successful management achieved here - through meticulous debridement, judicious use of 90% TCA, and defect restoration with Biodentine - highlights the value of an integrated surgical-endodontic approach in preserving tooth structure, function, and esthetics, even in advanced lesions. This case adds to the limited pool of literature on rare presentations of ECR and provides practical insights into tailoring treatment protocols for complex scenarios.

Presently, the follow-up period in this case is one year, and long-term results remain to be established. However, this case adds to the limited pool of literature on rare presentations of ECR and provides practical insights into tailoring treatment protocols for complex scenarios.

## References

[1] Bardini G, Orrù C, Ideo F, Nagendrababu V, Dummer P, Cotti E (2023). Clinical management of external cervical resorption: a systematic review. Aust Endod J.

[2] Heithersay GS (1999). Clinical, radiologic, and histopathologic features of invasive cervical resorption. Quintessence Int.

[3] Heithersay GS (2004). Invasive cervical resorption. Endod Top.

[4] Schwartz RS, Robbins JW, Rindler E (2010). Management of invasive cervical resorption: observations from three private practices and a report of three cases. J Endod.

[5] Mavridou AM, Bergmans L, Barendregt D, Lambrechts P (2017). Descriptive analysis of factors associated with external cervical resorption. J Endod.

[6] Patel S, Foschi F, Mannocci F, Patel K (2018). External cervical resorption: a three-dimensional classification. Int Endod J.

[7] Patel S, Lambrechts P, Shemesh H, Mavridou A (2018). European Society of Endodontology position statement: external cervical resorption. Int Endod J.

[8] Chen Y, Huang Y, Deng X (2021). External cervical resorption-a review of pathogenesis and potential predisposing factors. Int J Oral Sci.

[9] Talpos-Niculescu RM, Nica LM, Popa M, Talpos-Niculescu S, Rusu LC (2021). External cervical resorption: radiological diagnosis and literature review. Exp Ther Med.

[10] Kikuta J, Yamaguchi M, Shimizu M, Yoshino T, Kasai K (2015). Notch signaling induces root resorption via RANKL and IL-6 from hPDL cells. J Dent Res.

[11] Tronstad L (1988). Root resorption--etiology, terminology and clinical manifestations. Endod Dent Traumatol.

[12] Trope M (1998). Root resorption of dental and traumatic origin: classification based on etiology. Pract Periodontics Aesthet Dent.

[13] Mavridou AM, Hauben E, Wevers M, Schepers E, Bergmans L, Lambrechts P (2016). Understanding external cervical resorption in vital teeth. J Endod.

[14] Gunst V, Mavridou A, Huybrechts B, Van Gorp G, Bergmans L, Lambrechts P (2013). External cervical resorption: an analysis using cone beam and microfocus computed tomography and scanning electron microscopy. Int Endod J.

[15] Mavridou AM, Pyka G, Kerckhofs G (2016). A novel multimodular methodology to investigate external cervical tooth resorption. Int Endod J.

[16] Espona J, Roig E, Durán-Sindreu F, Abella F, Machado M, Roig M (2018). Invasive cervical resorption: clinical management in the anterior zone. J Endod.

[17] Chen Y, Huang Y, Deng X (2021). A review of external cervical resorption. J Endod.

[18] Spielman R, Ameh G, Brandes I, Berkowitz L, Elson N, Blum IR (2024). Challenges in differential diagnosis and treatment of cervical root resorption vs. root caries. Prim Dent J.

[19] Abuarqoub D, Aslam N, Jafar H, Abu Harfil Z, Awidi A (2020). Biocompatibility of Biodentine™ with periodontal ligament stem cells: in vitro study. Dent J (Basel).

[20] Butt N, Talwar S, Chaudhry S, Nawal RR, Yadav S, Bali A (2014). Comparison of physical and mechanical properties of mineral trioxide aggregate and Biodentine. Indian J Dent Res.

